# Hemozoin From the Liver Fluke, *Opisthorchis felineus*, Modulates Dendritic Cell Responses in Bronchial Asthma Patients

**DOI:** 10.3389/fvets.2019.00332

**Published:** 2019-10-16

**Authors:** Irina V. Saltykova, Wannaporn Ittiprasert, Kseniya V. Nevskaya, Yulia B. Dorofeeva, Natalia A. Kirillova, Evgeniy S. Kulikov, Vladimir V. Ivanov, Victoria H. Mann, Alexandra G. Pershina, Paul J. Brindley

**Affiliations:** ^1^Central Research Laboratory, Siberian State Medical University, Tomsk, Russia; ^2^Department of General Practice and Polyclinic Therapy, Siberian State Medical University, Tomsk, Russia; ^3^Department of Microbiology, Immunology and Tropical Medicine, Research Center for Neglected Diseases of Poverty, School of Medicine & Health Sciences, George Washington University, Washington, DC, United States

**Keywords:** Th1, immunoregulation, cytokine, asthma, dendritic cell, hemozoin, *Opisthorchis*

## Abstract

**Aims:** There is a general, inverse relationship between helminth infection and allergic diseases including bronchial asthma (BA). Proteins and other mediators released from parasitic worms exert cogent downmodulation of atopic and other allergic reactivity. We investigated the immune activities of an immortalized murine dendritic cell (mDC) line (JAWSII) and of primary human dendritic cells (hDCs) collected from study participants with and without BA after *Opisthorchis felineus* hemozoin (*Of*Hz) treatment.

**Methods and Results:** *in vitro*, expression of lymphocyte-activating factors—T helper 1 (Th1) induction and anti-inflammatory cytokines including tumor necrosis factor alpha (TNF-α), interleukin-1beta (IL-1β), IL-10, and IL-12β–increased significantly in mDCs pulsed with *Of*Hz. In parallel, primary dendritic cells (hDC) from cases clinically diagnosed with BA along with healthy controls were exposed *ex vivo* to *Of*Hz in combination with lipopolysaccharide (LPS). Whereas no significant change in the cellular maturation markers, CD83, CD86, and CD40, was apparent in BA vs. healthy hDC, pulsing hDC from BA with *Of*Hz with LPS induced significant increases in expression of IL-10 and IL-12β, although not of TNF-α or tumor growth factor-beta (TGF-β).

**Conclusions:** Liver fluke hemozoin *Of*Hz stimulated production of Th1 inducer and anti-inflammatory cytokines IL-10 and IL-12β from BA-hDC pulsed with *Of*Hz, an outcome that enhances our understanding of the mechanisms whereby opisthorchiasis contributes to protection against the atopic disease in liver fluke infection-endemic regions.

## Introduction

Helminth parasites establish chronic infections, characterized by modulation of both the innate and adaptive host immune response. A generalized, negative relationship between helminth infection and immune-related disorders is apparent for several disorders and helminth parasite species (1–3). Excretory–secretory (ES) products released from eukaryotic parasites are potent modulators of the immune response,which is central to the survival of these pathogens and for the maintenance of a chronic infection. There is active, sustained investigation of the mechanisms of the immunomodulation induced by helminths and the characterization of parasite-derived products with immunomodulation properties for treatment and prevention of the autoimmune-related diseases (4, 5). Indeed, investigation of the activities and properties of ES products has provided insights into the mechanisms of the host immune response modulation by parasites, which may be exploited for therapeutic intervention for allergic, autoimmune, metabolic, and inflammatory diseases (6–9).

Hemozoin (Hz) is an ES product with immunomodulatory properties known in a variety of eukaryotic parasites including *Plasmodium falciparum* (10), schistosomes (11–14), and liver flukes (15, 16). Hz is an inert, dark brown crystalline polymer of ferriprotoporphyrin IX produced by blood-feeding parasites. The synthesis of Hz by the parasite results in the detoxification of heme from digested blood. The effects of Hz purified from parasites [native Hz (nHz)] and of Hz synthesized (sHz) from hemin-synthetic Hz (β-hematin) have been investigated *in vitro* and *in vivo*. *In vitro*, dendritic cell (DC) and other cells such as monocytes phagocytize nHz (17). ES products of many helminths suppress DC maturation and modulate DC response to lipopolysaccharide (LPS) and other stimuli. Similar responses have been described following the incubation of DC with nHz purified from *P. falciparum* (*Pf* Hz). Exposure to *Pf* Hz impairs the maturation of immature DC (18). *Pf* Hz inhibits the loss of podosomes by DC and inhibits the upregulation of CD83 after stimulation by LPS, a potent activator of DC maturation (19). The addition of *Pf* Hz or sHz decreases production of LPS/IFN-promoted interleukin (IL)-12p70 [T helper 1 (Th1) responses development] and increases immune homeostasis cytokines including IL-10 and tumor necrosis factor alpha (TNF-α) from peripheral blood mononuclear cells (PBMCs) and CD14^+^ antigen-presenting cells (20).

Because the exposition and subsequent increased acceptance of the hygiene hypothesis, many diseases have been linked to exposure during childhood to microbial and eukaryotic pathogens, including helminth parasites (21–23). Asthma is a chronic inflammatory disease of the lung airways especially of the bronchi and bronchioles. Numerous factors contribute to asthma including environmental insults and allergens such as dust mites, pollen, and airborne pollutants from motor vehicles, along with a susceptible genetic phenotype. A rapid increase of incidence of asthma is evident in developed countries but is generally unapparent in less developed countries, especially in regions endemic for parasites. According to the hygiene hypothesis, the appearance of allergic diseases is inversely related to the decrease in prevalence of parasite infection, which can modulate the immune response and establish a host–parasite relationship and immunological milieu that accommodates both the helminth and its host (24–27). This provides a new area in which to search for intervention for the treatment and prevention of asthma.

Infection with the food-borne trematode *Opisthorchis felineus* is a highly prevalent liver fluke infection in Western Siberia, Russia (28, 29). Inverse relationships between *O. felineus* infection and allergy in a liver fluke endemic region have been reported (30). Epidemiological investigation has indicated that opisthorchiasis has a negative association with skin prick test reactivity (31). Infection with *O. felineus* diminishes the risk of atopic bronchial asthma (BA) associated with the *SOCS5* gene polymorphism (32). The treatment with filarial cystatin of human PBMCs from patients who are sensitive to timothy grass pollen caused a Th1 polarization, pointing to a potential therapy for asthmatics (33). Infection with *Schistosoma japonicum* and *Heligmosomoides polygyrus* can downregulate allergic airway inflammation (34, 35). However, given that intact infectious agents are likely to be pathogenic, parasite extracts such as *O. felineus* Hz (*Of*Hz) might offer a safer biotherapeutic. Accordingly, here, we addressed here whether *Of*Hz modulates DC immune function in patients during BA, a Th2 immune response-associated disease.

## Materials and Methods

### Hz Extraction and Purification

To extract Hz from *Opisthorchis felineus*, we used a slightly modified protocol based on methods reports for studies on host immunomodulation to Hz from *Schistosoma mansoni* (14) and *Plasmodium falciparum* (18). The purification of *Of*Hz involved the removal of host or parasite products adsorbed on the surface of *Of*Hz, which leads to variable and undetermined residue by decontamination of lipids and proteins. Briefly, *O. felineus* mature worms were collected from *O. felineus* metacercariae-infected hamster (50 metacercariae/hamster, Supplement 1). Pooled mature worms were washed three times with 1 × phosphate-buffered saline (PBS), and then 7 mL of packed parasites was final resuspended in PBS with 10 mL total volume. Worms were sonicated on ice until homogenized; the lysate was subjected to centrifugation at 1,000 × g for 60 s at room temperature. The upper aqueous contents of the supernatant were transferred to a new tube, and the pellet containing the Hz was collected after centrifugation at 8,000 × g for 20 min. Pelleted Hz was resuspended in PBS and then subjected to precipitation in chloroform, methanol, and water (36). Pelleted Hz was resuspended in 2 mL of PBS, briefly sonicated on ice, and exposed to 1% proteinase K at 37°C for 18 h. Subsequently, the Hz was re-pelleted at 10,000 × g for 20 min and washed sequentially three times in PBS containing 2% sodium dodecyl sulfate (SDS), three times in 0.1 M of NaHCO_3_ (pH 9.1) containing 2.5% SDS, and five times in distilled water. The contamination of Hz samples was assessed for proteins by silver-stained SDS–polyacrylamide gel electrophoresis (PAGE) and for nucleic acids by use of ethidium bromide-stained agarose gels. The absence of *O. felineus* egg contamination was confirmed by light microscopy.

Levels of endotoxin level were determined using an endpoint chromogenic *Limulus* amebocyte lysate assay (Lonza AG, Visp, Switzerland,). A standard curve was established using a range of concentrations of hematin diluted in 100 mM of NaOH, 2% SDS, and 3 mM of EDTA, for which absorbance at 401 nm was measured using a spectrophotometer. To determine the concentration of *Of*Hz, the absorbance at 401 nm of aliquots of *Of*Hz dissolved in 100 mM of NaOH, 2% SDS, and 3 mM of EDTA was compared with the standard curve.

### DC Stimulation—Exposure of JAWSII Cells to *Of*Hz

The semi-adherent JAWSII cell line, a granulocyte-macrophage colony-stimulating factor (GM-CSF)-dependent dendritic (DC) line established from bone marrow cells of p53-knockout C57Bl/6 mouse, was purchased from the American Type Culture Collection (CRL-1194; ATCC, Manassas, VA). JAWSII cells were maintained in complete Roswell Park Memorial Institute medium (RPMI) 1640 culture with 4 mM of l-glutamine, HEPES (Thermo Fisher Scientific) consisting of 10% (v/v) fetal bovine serum (FBS), 5 ng/mL of GM-CSF, 10 U/mL of penicillin and 100 μg/mL of streptomycin, 0.5 mM of 2-ME, and 1 mM of sodium pyruvate, in 5% CO_2_ in air at 37°C. Cultures were maintained by transferring nonadherent cells to a centrifuge tube and treating attached cells with 0.25% trypsin−0.03% EDTA (Gibco) at 37°C for 5 min, followed by pooling the two populations of cells together and dispensing into new flasks and/or for downstream analysis. To stimulate JAWSII cells, 300,000 cells/mL were seeded into wells of 6-well plates for 24 h before exposure to liver fluke Hz, *Of*Hz. The cells were divided into three groups: control (no treatment), mock (1 × PBS treatment), and *Of*Hz-treated (100 nm of *Of*Hz in 1 × PBS). JAWSII cells were pulsed with *Of*Hz for 48 h and harvested as above. The cells were washed three times with 1 × PBS before proceeding to RNA extraction.

### RNA Extraction and Quantitative Reverse Transcriptase Polymerase Chain Reaction (RT-PCR) of Anti-inflammatory and Inflammatory Cytokine

Total RNA was extracted from JAWSII and *Of*Hz-treated JAWSII cells using the RNAzol RT reagent (Molecular Research Center, Inc., Cincinnati, OH), which removes contaminated DNA (37), and its concentration and purity were determined using the NanoDrop ND-1000 spectrophotometer (OD260/280, ~2.0). RNAs were reverse transcribed into cDNA using iScript Reverse Transcript (Bio-Rad). First-strand cDNA was performed RT-qPCR using SsoAdvanced Universal SYBR Green Supermix (Bio-Rad) and run in triplicate using the iQ5 Real time PCR thermal cycler (Bio-Rad); thermal cycling was as follows: initial denaturation at 95°C for 30 s, 40 amplification cycles each consisting of denaturation at 95°C for 15 s, annealing at 60°C for 30 s, and final heating at 60–95°C to obtain the melting curve. Samples were run in triplicates after which the output was analyzed using the iQ5 software (Bio-Rad). The delta-delta Ct method with normalization to mouse GAPDH expression (38) was used to calculate the relative expression of the IL-1β, IL-10, IL-12b, and TNF-α genes. The data were expressed as differential fold change compared with the control group, with fold changes reported as mean and 95% confidence interval (CI) of difference. The statistical significance of cytokine transcript induction from *Of*Hz-treated groups were compared with control groups using two-way analysis of variance (ANOVA), followed by Dunnett's multiple comparison test (Prism software).

### Monocyte-Derived DCs

Purification of human PBMCs was performed using a two-step gradient centrifugation (39). Briefly, peripheral blood was diluted with Hank's balanced salt solution (HBSS) (1:1), loaded on a Ficoll–Hypaque gradient (Sigma, Moscow, Russia), and centrifuged for 30 min at 600 × g at room temperature. PBMCs were collected, washed twice in HBSS (pH 7.4), resuspended in serum-free RPMI, and mixed with 1.5 × volume of isotonic Percoll solution (IPS) (Percoll:PBS, 9:1 v/v, *p* = 1.123 g/mL). Thereafter, PBMCs were overlaid carefully with Percoll–RPMI solution 1 (IPS:RPMI, *p* = 1.064 g/mL) and Percoll–RPMI solution 2 (IPS:RPMI, *p* = 1.032 g/mL). Monocytes were collected from the RPMI–Percoll interface after centrifugation at 2,000 × g for 50 min at 20°C. The purity of monocytes was 79–87% as determined by flow cytometric quantification of CD14^+^-positive cells. Monocytes were seeded into 24-well plates at 0.5–1 × 10^6^ cells/well and cultured for 96 h in complete RPMI 1640 medium supplemented with 10% heat-inactivated FBS (HyClone, Thermo Fisher Scientific,), 50 μM of β-mercaptoethanol, 110 mg/L of 2 mM of l-glutamine, 1 × penicillin–streptomycin (PanEco, Russia) in the presence of GM-CSF (100 ng/mL), and IL-4 (50 ng/mL) (Sigma) in 5% CO_2_ in air at 37°C. Maturation of DC was induced by pulsing with LPS; immature DCs were incubated with LPS (100 ng/mL) and LPS plus *Of*Hz (15 μg/mL). DCs were harvested 36 h later and supernatants collected for immunoassays; aliquots of supernatants were stored at −80°C for cytokine analysis.

### Immunoassays for DC Markers and Secreted Cytokines

The levels of the co-stimulatory and maturation markers CD40, CD83, and CD86 on human DCs (40) were analyzed by flow cytometry using antibodies from BD Biosciences, San Diego, CA. Cells (10^4^) were monitored using the Accuri C6 flow cytometer (Becton Dickinson, Franklin Lakes, NJ), and the data analyzed using the Cell Quest software (BD Biosciences). Levels of IL-10, IL-12β, TNF-α, and tumor growth factor-beta (TGF-β) were measured by enzyme-linked immunosorbent assay (ELISA) (eBioscience, Thermo Fischer Scientific).

### Study Participants Diagnosed With BA

The Ethics Committee of the Siberian State Medical University, Russia, approved the study, with approval number 4815. All participants provided written consent. Blood from 10 cases with severe BA and nine healthy volunteers (personnel at the Siberian State Medical University, Russia) was examined in this study. The elderly asthma patients (>58 years old), eight females and one male, were diagnosed as BA (Table 1) according to the Global Initiative for Asthma criteria (41), using a standardized questionnaire and results of physical and laboratory examinations for more than 20 years. Non-atopic and non-smoking individuals without a family history of asthma/allergy were included as healthy controls. All of the BA cases and the healthy controls were negative for *O. felineus* infection, by stool examination, at the time of blood collection. Table 1 outlines the epidemiological characteristics and other records of the participants.

**Table 1:** Characteristics of study participants.

	**Bronchial asthma (*n* = 10)**	**Control (*n* = 8)**
Age in years	64.5 ± 5.7	20.6 ± 1.4
Females/males	9/1	7/2
Duration of asthma (years)	20.7 ± 9.1	NA
FEV1 (%)	64.2 ± 7.8	NA
Emergency medical care in the past year	2.6 ± 1.2	None
PC20 (mg/mL)	0.06 ± 0.0	None

*Data presented as mean ± SD. FEV1, forced expiratory volume per second (FEVI); PC20, histamine provocative concentration causing a 20% fall in FEV1*.

### RNA Extraction From Human DCs and Differential Cytokine Gene Expression

Total RNAs were isolated from non-stimulated human DCs and from DCs stimulated with Hz using the TRIzol (Invitrogen, USA) extraction method followed by purification on columns (Qiagen, UK). Total RNA was reverse transcribed into cDNA following the protocol for RevertAid First-Strand cDNA Synthesis Kit (Thermo Fisher). The cDNAs were amplified using PCR with gene-specific primers designed by using CLC Main Workbench 7.0 (CLC Bio, Aarhus, Austria). Each PCR was carried out in duplicate with optimized primer concentrations using qPCRmix-HS SYBR (Evrogen, Russia) in a thermal cycler CFX-96 (Bio-Rad), with the following thermal cycling conditions: one cycle of 10 min denaturation at 95°C; 34–37 cycles of 1 min at 94°C, 1 min at 53–57°C, and 1 min at 72°C; and a final extension step at 72°C for 10 min. A dissociation curve was included in each run to ensure specificity of amplification. Beta-actin (*ACTB)* was used as the reference gene. The relative expression of genes was calculated using delta-delta Ct method; data were expressed as fold change compared with the untreated group (38).

### Statistics

Data analysis was accomplished with the assistance of the Prism 6, GraphPad software. Cell culture data were analyzed by two-way ANOVA with Dunnett's multiple comparison test. When the variables did not show normal distribution, they were compared using the Mann–Whitney *U*-test (two-tailed). The Wilcoxon matched pairs test was applied to dependent samples (*P* ≤ 0.05 were considered to be statistically significant.

## Results

### Chemokine Transcript Profiles in JAWSII After *Of*Hz Stimulation

**Figure 1:**
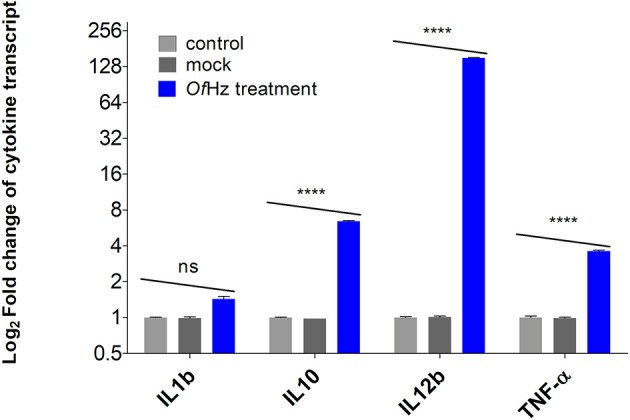
Log2-transformed difference fold changes in cytokine mRNA levels [*Of*Hz treated (blue color bar)/JAWSII control cells (gray color bars)] after GAPDH normalization. Error bars represent mean and 95% CI of difference (*n* = 3). *Of*Hz, *Opisthorchis felineus* hemozoin. ****P ≤ 0.001.

The transcript levels encoding IL-1β, IL-10, IL-12β, and TNF-α were analyzed after JAWSII cells were pulsed with 100 nm of *Of*Hz in PBS for 48 h. Whereas, differences in expression of IL-1β in *Of*Hz-treated and control groups (mock and no treatment) were not seen, there were statistically significant increases in transcription of IL-10, IL-12β, and TNF-α by 6.4-, 151-, and 3.6-fold, respectively (Figure 1).

### Maturation of DCs Induced by LPS Was Unaffected by *Of*Hz

**Figure 2:**
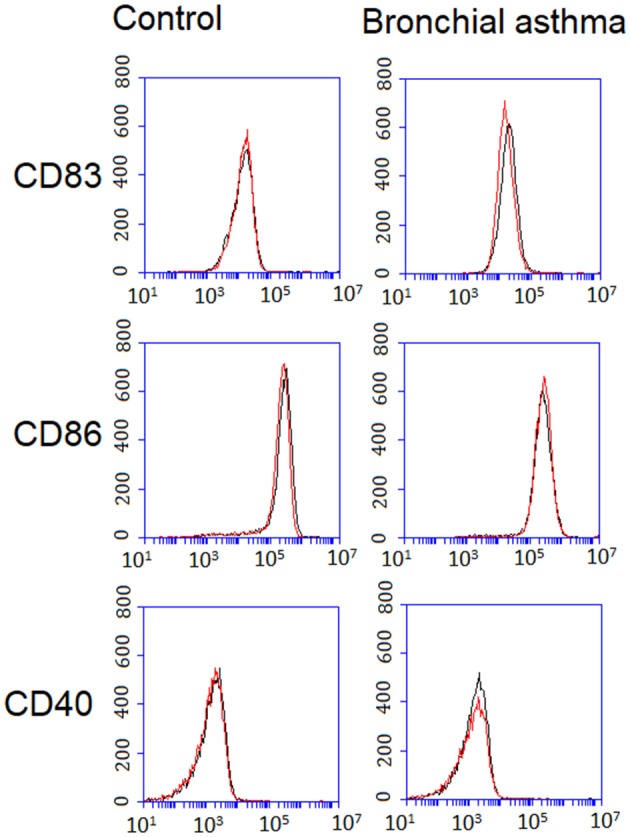
Expression of CD83, CD86, and CD40 on DCs from control and bronchial asthma groups. Mature DC phenotype was analyzed after LPS along stimulation (Hz–) (black line) and stimulation with LPS and *Of*Hz combination (Hz+) (red line). Expression of CD83, CD40, and CD86 was unaffected by *Of*Hz in DCs activated by LPS. DCs, dendritic cells; Hz, hemozoin; LPS, lipopolysaccharide.

The effect of *Of*Hz on maturation of DCs in response to LPS was investigated by analysis of hallmark markers of the mature DC phenotype (40). Monocyte-derived DCs from participants with BA and controls were stimulated with LPS alone and in combination with *Of*Hz. CD83, CD40, and CD86 expression on DCs were measured. Expression of CD83, CD40, and CD86 was unaffected by *Of*Hz in DCs activated by LPS (Figure 2, Table S1).

### Change of DCs Cytokines Expression and Secretion Related to *Of*Hz Treatment

**Figure 3:**
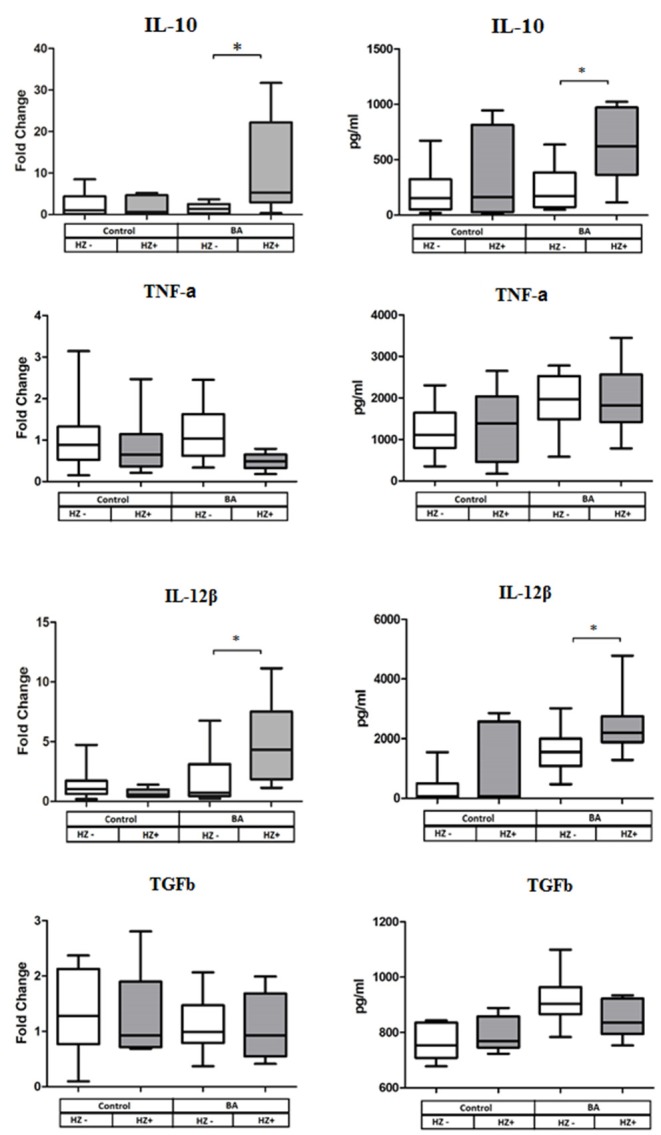
Cytokine gene expression (left panel of the figure) and cytokine secretion (right panel) by human DC after LPS along stimulation (Hz–) and stimulation with LPS and *Of*Hz combination (Hz+). Data are presented as medians (Q1–Q3); the Wilcoxon matched pairs test was applied for dependent samples. BA, bronchial asthma group; DCs, dendritic cells; LPS, lipopolysaccharide; Hz, hemozoin; *Of*Hz, *Opisthorchis felineus* hemozoin. *P ≤ 0.05.

DCs from BA cases exposed *in vitro* to *Of*Hz exhibited significant upregulation of expression of *IL10* and *IL12b*. In addition, we measured the levels of IL-10, TGF-β, TNF-α, and IL-12β by ELISA in culture supernatants. Concentrations of IL-10 and IL-12β were increased in supernatants of the DC from the BA cases treated with *Of*Hz in comparison with non-*Of*Hz-treated DCs from BA cases. Thus, the IL-10 and IL-12b gene expression and ELISA data were in concordance. By contrast, treatment of DC donated by healthy controls with *Of*Hz failed to affect cytokine gene expression and secretion (Figure 3).

## Discussion

Synthetic Hz has been investigated as an adjuvant in the anti-allergen vaccines. Atopic dermatitis that develops in beagles is similar to that seen in humans and is associated with elevated titers of IgE antibodies against the house dust mite allergen, Derf2. Immunization of dogs with Derf2 together with alum and/or sHZ, with subsequent sensitization by the allergen, provoked significantly elevated levels of IgG2, but not IgG1, antibodies in the sHz-treated group that resembled a Th1-like immune response (42). Treatment of PBMCs from healthy, malaria-free donors with sHz induced increases in IL-12p40 and IL-10 transcripts at 24 h of exposure with further stabilization of the expression levels of the cytokines relative to control conditions (43). Much less in known about properties of Hz isolated from blood-feeding helminths.

This is the first report to investigate the effect of liver fluke Hz on immature mouse DC cytokine expression level and immunomodulation activity on human DC maturation and cytokine expression and secretion. Specifically, the study investigated cytokine responses in an immature DC, JAWSII cells, following *Of*Hz stimulation to estimate *Of*Hz immunomodulation activity. Second, using leads from the responses of these JAWSII monocytes, we investigated the ability of stimulation with *Of*Hz to affect LPS-induced maturation of human DCs and the consequent cytokine responses. Two groups of participants were included in the study to isolate monocytes for DCs generation: healthy volunteers and BA adults. Initially, exposure to *Of*Hz induced expression of IL-12β, IL-10, and TNF-α in JAWSII. Immune-activating and immune-suppressive effects have been reported for Hz in experiments *in vitro*.

DCs play critical roles in determining T-cell differentiation in the context of allergen exposure (23). Marked differences were evident between asthma and healthy control groups in the expression and release of cytokines by DCs in response to co-incubation with *Of*Hz and LPS. *Of*Hz potentiated the upregulation by LPS of cytokines IL-10 and IL-12β by human DC but only in the asthma group. There was no clear trend in IL-12β production by DCs in the asthma-free group in response to *Of*Hz. In general, we observed the variable individual responses in cytokine production by DCs after *Of*Hz exposure among the healthy control participants. Cell immunophenotypic characteristics can influence diverse responses to stimulation by Hz. Fibronectin incubated at physiologic concentrations with fibronectin-free Hz binds with high affinity to Hz. The addition of fibronectin-containing Hz to adherent monocytes induces rapid stimulation of reactive oxygen species production and increase of TNF and monocyte chemotactic protein 1 by human monocytes. These responses arise from the interaction of fibronectin with fibronectin-receptors TLR4 and integrin CD11b/CD18 (44). TLR4 plays a key role in the allergic inflammation and severity of asthma (45).We hypothesize that the prevalence of the fibronectin receptors, notably TLR4, on the DC surface impairs the effects of stimulation by *Of*Hz.

Mature DCs secrete IL-10, IL-12, and proinflammatory cytokines, and these cytokines are known to participate in T-cell differentiation. *Of*Hz stimulation induced IL-12b, IL-10 expression by immature DC and potentiated LPS-induced expression and production of IL-10 and IL-12β by human DCs in the BA asthma group. Asthma is a Th2-associated inflammatory disease with increased levels of IgE and Th2 cytokines. IL-12 as Th1-promoting cytokine has potential roles in the antagonism of Th2 cytokine responses and IgE synthesis that prevents the progress of airway inflammation in asthma (46). IL-10 has immunosuppressive properties, and induction of IL-10 by helminths is considered as one of the possible mechanisms of parasite survival in the anti-inflammatory environment (47). The production of Th1-promoting cytokine IL-12β and anti-inflammatory cytokine IL-10 by asthma DCs upon *Of*Hz stimulation can suggest a role for Hz released during infection with *Opisthorchis felineus* in the Th1/Th2 balance regulation.

To extend the findings related to *Of*Hz-activated cytokine expression in DC and to determine whether *Of*Hz may be immunomodulatory, maturation markers, cytokine expression, and secretion by human DCs were investigated. *Of*Hz did not appear to affect LPS-induced upregulation of key markers associated with a DC mature phenotype, including CD83, CD86, and CD40. It is noteworthy that exposure with crude *O. felineus* extract leads to the downregulation of costimulatory molecules CD83 and CD86 in LPS-induced DCs, generated from monocytes of asthma patients (48). Accordingly, *Of*Hz may be considered as a mediator that plays a key role in the *O. felineus*-derived immune response. Further investigation of the local liver immune cell response to secretion and accumulation of *Of*Hz in bile ducts might provide insights into the chronic inflammation and other hepatic morbidity due to opisthorchiasis. Moreover, investigation of *Of*Hz in asthma model *in vivo* can be expected to increase our understanding of the potential benefits of treatment with *Of*Hz for the treatment of asthma.

In conclusion, our data demonstrate that *Of*Hz induces expression of Th1 cytokines in immature mouse DC and that *Of*Hz potentiates LPS-induced expression and production of IL-10 and IL-12β by DC in asthma patients. These results complement earlier findings that demonstrated the regulation of the host immune response by the helminth ES products, which play a protective role against allergy and allergic diseases (49), including findings in regions endemic for *O. felineus*, and that demonstrated the inverse relationship between opisthorchiasis and allergic diseases (31).

## Data Availability Statement

The datasets generated for this study are available on request to the corresponding author.

## Ethics Statement

The studies involving human participants were reviewed and approved by The Ethics Committee of the Siberian State Medical University, Russia. The patients/participants provided their written informed consent to participate in this study. The animal study was reviewed and approved by The Ethics Committee of the Siberian State Medical University, Russia.

## Author Contributions

IS conceived and planned the experiments, analyzed the data, contributed to the interpretation of the results, and wrote the manuscript with input from all authors. WI performed the experiments, analyzed the data, and wrote the manuscript with input from all authors. YD, KN, WI, and VI performed the experiments. VI were involved in planning and supervised the work. EK and NK contributed to patients sample collection. AP and VM provided critical feedback and helped shape the research. PB supervised the project and wrote the manuscript.

### Conflict of Interest

The authors declare that the research was conducted in the absence of any commercial or financial relationships that could be construed as a potential conflict of interest.
