# Supplementary material for: Hemozoin From the Liver Fluke, Opisthorchis felineus, Modulates Dendritic Cell Responses in Bronchial Asthma Patients
Source: Front Vet Sci. 2019 Oct 16;6:332. doi: 10.3389/fvets.2019.00332 (PMC6843058; doi:10.3389/fvets.2019.00332)
Supplement: Supplementary file 1 [file Table_1.DOCX]

**Supplementary file**

**Animal model of infection**

*O. felineus* metacercariae were obtained from naturally infected freshwater ﬁshes (Leuciscus leuciscus, Rutilus rutilus) caught in the river in the endemic area of Western Siberia (the river Tom, Tomsk region, Russia).

Animals were obtained from the SPF-vivarium of the Institute of Cytology and Genetics, Siberian Branch, Russian Academy of Sciences, Novosibirsk. The animals were infected intragastrically with 50 metacercariae per hamster. The hamsters were housed two to a cage (OptiRAT) under conventional conditions and were permitted ad libitum access to food and water. After 10 weeks post-infection, the animals were euthanized in a CO2 chamber. Adult worms were obtained from bile ducts, washed with PBS and proceed for Hz extraction and purification.

**S Table 1**. Expression of CD markers of human DCs after exposure to LPS or LPS in combination with *Of*Hz.

|  | **Bronchial asthma**  **LPS** | **Bronchial asthma**  **LPS+*Of*Hz** | **Control**  **LPS** | **Control**  **LPS+*Of*Hz** |
| --- | --- | --- | --- | --- |
| **CD83+** | 16.25 (13.38- 21.60) | 12.90 (9.62- 21.83) | 20.50 (17.20- 24.35) | 20.10 (15.05- 26.30) |
| **CD86+** | 98.85 (97.53- 99.43) | 99.25 (98,73- 99.50) | 98,60 (95,00- 99.15) | 98.80 (95.95- 99) |
| **CD40+** | 7.8 (4.4- 21.1) | 8.3 (7-13.1) | 2.8 (2.15-6.1) | 3.6 (2.3-6.8) |

Data presented as medians (Q1–Q3), %, The Wilcoxon matched pairs test was applied for dependent samples.
